# What do pediatric surgeons think about sexual issues in dealing with patients with anorectal malformations? The ARM-Net consortium members’ opinion

**DOI:** 10.1007/s00383-019-04506-0

**Published:** 2019-07-05

**Authors:** Eva Elisa Amerstorfer, Caterina Grano, Chris Verhaak, Araceli García-Vasquez, Marc Miserez, Annemone Radleff-Schlimme, Nicole Schwarzer, Michel Haanen, Ivo de Blaauw, Ekkehart Jenetzky, Alida van der Steeg, Iris A. L. M. van Rooij, ARM-Net consortium, Dalia Aminoff, Piero Bagolan, Barbara Iacobelli, Paul Broens, Stefan Deluggi, Johanna Ludwiczek, Maria Fanjul, Francesco Fascetti-Leon, Piergiorgio Gamba, Carlos Giné, Stefano Giuliani, Jan Goseman, Martin Lacher, Sabine Grasshoff-Derr, Stefan Holland-Cunz, Ernesto Leva, Anna Morandi, Gabriele Lisi, Omid Madadi-Sanjan, Igor Makedonsky, Carlo Marcelis, Paola Midrio, Onur Ozen, Alessio PiniPrato, Carlos Reck-Burneo, Heiko Reutter, Stephan Rohleder, Inbal Samuk, Eberhard Schmiedeke, Pim Sloots, Herjan van der Steeg, Pernilla Stenström, Holger Till, Patrick Volk, Tomas Wester

**Affiliations:** 1 Department of Pediatric and Adolescent Surgery, Medical University of Graz, Auenbruggerplatz 34, 8036 Graz, Austria; 2 Department of Psychology, Sapienza University of Rome, Rome, Italy; 3 Department of Medical Psychology, Radboudumc Nijmegen, Nijmegen, The Netherlands; 4 Department of Pediatric Surgery, University Hospital, 12 de Octubre, Madrid, Spain; 5 Department of Abdominal Surgery, University Hospital Gasthuisberg, KU Leuven, Belgium; 6 German Self-help Organization for Anorectal Malformations SoMA e.V., Munich, Germany; 7 VA-Dutch Patient Organization for Anorectal Malformations, Huizen, The Netherlands; 8 Department of Surgery-Pediatric Surgery, Amalia Children’s Hospital-Radboudumc Nijmegen, Nijmegen, The Netherlands; 9 Department of Child and Adolescent Psychiatry, University Medical Center of the Johannes Gutenberg University, Mainz, Germany; 10 Institute of Integrative Medicine, Witten/Herdecke University, Herdecke, Germany; 11 Department of Pediatric Surgery, Emma Children’s Hospital, AMC and VU University Medical Center, Amsterdam, The Netherlands; 12 Department for Health Evidence, Radboud Institute for Health Sciences, Radboudumc Nijmegen, Nijmegen, The Netherlands

**Keywords:** Anorectal malformation, Sexuality, Sexual functioning, Adolescence, Training, ARM-Net

## Abstract

**Purpose:**

Since pediatric surgeons aim to follow their patients with anorectal malformations (ARM) into adulthood the aim of this study was to investigate how pediatric surgeons deal with sexual issues related to ARM.

**Methods:**

In 2018, a questionnaire was developed by the working group “Follow-up and sexuality” of the ARM-Net consortium and sent to all consortium-linked pediatric surgeons from 31 European pediatric surgical centers. Obtained data were statistically analyzed.

**Results:**

Twenty-eight of 37 pediatric surgeons (18 males/10 females) answered the questionnaire. The majority of pediatric surgeons (82%) think they should talk about sexual issues with their patient. More than 50% of pediatric surgeons do not feel at all or only moderately confident discussing the topic of sexuality. Most pediatric surgeons require more support (96%) and wish to be trained in sexuality and sexual issues (78%) to feel confident towards their ARM-patients/parents. For optimal care, sexual issues with ARM-patients should be managed by a multidisciplinary team.

**Conclusions:**

Pediatric surgeons feel that sexuality is an important issue for their ARM-patients, which they are primarily responsible of but should be managed in concert with a multidisciplinary team. A training in sexuality is wished to feel more confident about this specific issue.

## Introduction

Sexual development already starts in childhood, when toddlers start to explore their body and interact with other children by inducing games such as “doctor” and “patient”, building up their sexual identity [1]. An impaired body image, as well as impaired self-esteem, problems with incontinence, dependence on parental help and other psychosocial factors may interfere with normal sexual development during childhood and adolescence [2–5]. These factors might be the reason that a significant number of adult patients with an anorectal malformation (ARM) is dissatisfied with their partnership and sexuality [5, 6] or report some forms of sexual dysfunction [7, 8]. Patients with an ARM undergo reconstructive surgeries during infancy or childhood and often suffer from remaining perineal or abdominal scars as well as sustained fecal and urinary incontinence or constipation. Depending on the degree of symptoms, quality of life (QoL), social life, psychological morbidity, as well as sexual health are affected in this patient cohort during childhood, adolescence and adulthood [4, 5, 9–13]. Though, not only might sexuality itself be a major issue to patients with ARM, it has been further stated that in adult male ARM patients difficulties in close and sexual relationships are predictive for depressive feelings [14]. Thus, as it has been stressed by others, it is paramount that beside anatomic reconstruction, sexual function is preserved and that ARM patients can achieve independence from their parents, become self-efficient and are able to establish contact with peers to develop satisfying and prosperous intimate and sexual relationships [4, 5].

The precise anatomic reconstruction and preservation of organic function are the primary targets of pediatric surgeons in treating patients with ARM. Nowadays, many pediatric surgeons follow their patients into adulthood and experience problems in the transfer from adolescent to adult care regarding sexual health. In that context, an interview by sexologists with adolescents with ARM revealed that the patients wished to be contacted and informed about possible sexual problems related to their disease by their pediatric surgeon during follow-ups [13]. However, as demonstrated by a recent study, only a few patients are educated about sexual issues related to ARM during medical care, although more than half of the investigated patients would have appreciated that [7]. Talking freely about sexual issues, and evaluating sexual function, however, represents a peculiar situation for patients and their physicians. Reasons for infrequent communication may include marginal training of surgeons on intimate relationships and sexual issues and hesitancy of the patients due, for example, to potential embarrassment [15].

The goal of the present study was therefore to investigate how pediatric surgeons deal with sexual issues related to ARM. More specifically, we were interested in understanding if pediatric surgeons feel that they should talk of sexual issues with their ARM patients, if they feel confident in doing that, if their confidence differs depending on the patient’s gender, at what age sexual issues should be discussed with the patient, if they wished to be more trained in sexuality and sexual issues, and which professionals they think should be included in managing patients with ARM during their sexual development.

## Methods

In 2010, several European pediatric surgical centers involved in the care for patients with ARM founded the ARM-Net consortium, involving pediatric surgeons, epidemiologists, psychologists, geneticists, and representatives of patient organizations [16]. The aim of this consortium is to exchange data and knowledge to improve clinical care and to perform research into genetic, epidemiological and clinical subjects as well as registering and monitoring frequencies of types of ARM, additional malformations, surgical procedures, complications, and outcomes. In addition, its focus is to harmonize diagnostic classification, possible interventions, and follow-up to improve QoL of ARM patients.

### Questionnaire

A questionnaire evaluating sexual issues was developed by the working group “Follow-up and sexuality” of the ARM-Net consortium based on analyses of previous literature conducted in the field of sexuality in ARM patients, and on relevant information given by pediatric surgeons and by patients and parents associations represented in the ARM-net consortium. The final questionnaire was composed of 21 items. Examples of items of the questionnaire were: “Do you address questions about sexual issues to your patients during follow-up?” answered on a dichotomous Yes/No scale or “Do you feel prepared to talk about sexuality with your ARM patients?” answered on a five-point response scale ranging from one (not at all) to five (extremely).

### Procedures

All the pediatric surgeons (*N* = 37) belonging to the ARM-Net consortium received an e-mail containing a weblink with the final online version of the questionnaire implemented through the Survey monkey provider. Once the link was opened, a brief presentation of the study was given and participants were asked to give their informed consent, before starting filling in the questionnaire. The questionnaire was sent by e-mail a first time in March 2018 with a first recall in April and a last recall in September of the same year.

### Statistical analyses

All the questionnaires were exported from the Survey monkey provider as an Excel file. The database was cleaned (eliminating duplicates and empty cases) and transformed in a SPSS file. The program IBM Statistics SPSS 25 was used to analyze the data. Descriptive statistics [mean values, standard deviations (SD), or if more appropriate median and range, and percentages] were calculated. For some questions the percentages are provided for male and female pediatric surgeons separately to consider possible different perspectives.

## Results

Of the 37 pediatric surgeons contacted, 28 answered the questionnaire for a response rate of 76%. A total of 24 pediatric units participated in the study, from four units two pediatric surgeons participated. The respondents came from 11 different European countries, namely Austria, Belgium, Germany, Israel, Italy, The Netherlands, Spain, Sweden, Switzerland, Ukraine, and United Kingdom (Table 1). More males than females participated and the mean age at filling in the questionnaire was 47 years. The number of patients the pediatric units reported to see each year at their clinic ranges from 15 to 250 of which 3–30 are new patients. The number of adolescent or adult patients is much lower and ranges from 2 to 60 and approximately 25% of them are new each year and the rest is followed-up.

**Table 1:** Characteristics of the participating pediatric surgeons and pediatric units

Gender pediatric surgeons, *N* (%)	
Male	18 (64.3)
Female	10 (35.7)
Age pediatric surgeon, mean (SD)	46.9 (7.7)
Number of patients seen per year in a pediatric unit, median (range)	
Total number of ARM patients	55 (15–250)
Number of adolescent/adult ARM patients	7 (2–60)
Number of new ARM patients	8 (3–30)
Number of new adolescent/adult ARM patients	2 (0–15)
Number of pediatric units per country, *N*/number of patients seen each year, mean (SD)	
Austria	2/64 (51)
Belgium	1/15
Germany	4/29 (15)
Israel	1/missing
Italy	5/136 (87)
The Netherlands	3/152 (84)
Spain	3/75 (35)
Sweden	2/80 (87)
Switzerland	1/25
Ukraine	1/20
United Kingdom	1/20

*ARM* anorecal malformation, *SD* standard deviation

Results from the questions asked to the pediatric surgeons about their current practice on the issue of sexuality are reported in Table 2. Most ARM patients are followed-up into their adolescence. In 54% pediatric surgeons screened for sexual function from infant age onwards, but only three of them used standardized questionnaires for this. Of these three, one used the Mathtech questionnaire [17] and two used a questionnaire developed by themselves, one of them derived from a general national questionnaire for adolescents that was adapted for ARM patients. Half of the participating pediatric surgeons asked questions about sexual experiences to adolescent or adult ARM patients and 39% of the patients talked about sexuality in general, but none of them often. There is no difference found between male or female pediatric surgeons on these issues. In most pediatric units (82%) a psychologist was part of the team, working with ARM patients in the hospital, and in 50% these psychologists were dedicated to ARM patients. A sexologist was only present in the team in one-fifth of all pediatric units and was often not dedicated to ARM patients.

**Table 2:** Current practice on the issue of sexuality in the care of ARM patients by pediatric surgeons

	*N* (%)^a^
Do you follow your ARM patients into their adolescence?	
Yes, all of them	15 (57.7)
Yes, most of them	9 (34.6)
Yes, but only a small part of them	2 (7.7)
No	0 (0.0)
Do you screen for sexual function^b^ from infant age (1 year of age) onwards?	
Yes	15 (53.6)
No	13 (46.4)
Do you use any standardized questionnaires for asking about sexuality?	
Yes	3 (10.7)
No	25 (89.3)
Do you address questions concerning sexual experiences to your adolescent/adult ARM patients during follow-up visits?	
Never	1 (3.6)
Seldom	12 (42.9)
Sometimes	8 (28.6)
Often	5 (17.9)
Very often	2 (7.1)
Do your ARM patients ask you questions about sexuality?	
Never	5 (17.9)
Seldom	12 (42.9)
Sometimes	11 (39.3)
Often	0 (0.0)
Very often	0 (0.0)
Do you have a psychologist in your team at the hospital?	
Yes	23 (82.1)
No	5 (17.9)
If yes,	
Is he/she dedicated to the ARM patients?	
Yes	12 (52.2)
No	11 (47.8)
Is he/she a pediatric psychologist?	
Yes	16 (72.7)
No	1 (4.5)
I don’t know	5 (22.7)
Do you have a sexologist in your team at the hospital?	
Yes	6 (21.4)
No	22 (78.6)
If yes,	
Is he/she dedicated to the ARM patients?	
Yes	1 (16.7)
No	5 (83.3)
Does he/she work also with children/adolescents?	
Yes	4 (66.7)
No	2 (33.3)

*ARM* anorectal malformation

^a^Percentages are calculated from those who responded to the question, so ignoring missing answers

^b^For example erection in neonatal age, secondary characteristics during puberty

The majority of pediatric surgeons specified that the issue of sexuality is important for ARM patients and that they should talk about this issue with their patients (Table 3). A smaller group of surgeons, but still 64% thinks that they should be the first responsible ones to address sexual function with patients, with a difference in opinion between female (80%) and male pediatric surgeons (56%). The age at which to start to discuss questions about sexuality was suggested to be between 11 and 17 years, with a mean of 14.6 years (SD:1.6). The idea of 50% of the pediatric surgeons is that adolescent or adult patients would like to talk about sexuality with them often or very often if they were given the opportunity. The same idea of the pediatric surgeon is true for the parents talking about the sexuality of their child.

**Table 3:** Opinions about practice on the issue sexuality in ARM patients by pediatric surgeons

	*N* (%)^a^
Do you think sexuality is an important issue for ARM patients?	
1—not at all important	0 (0.0)
2	1 (3.6)
3	1 (3.6)
4	8 (28.6)
5—extremely important	18 (64.3)
Do you think the pediatric surgeon should talk about sexual issues with his/her patients?	
Yes	23 (82.1)
No	5 (17.9)
Do you think, if they were given the possibility, your adolescent or adult patients/their parents would like to talk more about sexuality with you?	Patients/parents
Never	0 (0.0)/0 (0.0)
Seldom	4 (14.3)/5 (17.9)
Sometimes	10 (35.7)/7 (25.0)
Often	12 (42.9)/15 (53.6)
Very often	2 (7.1)/1 (3.6)
Do you feel prepared to talk about sexuality with your ARM patients/their parents?	Patients/parents
Not at all	2 (7.1)/2 (7.1)
A little	7 (25.0)/6 (21.4)
Moderately	6 (21.4)/7 (25.0)
Quite well	13 (46.4)/12 (42.9)
Extremely	0 (0.0)/1 (3.6)
How much confidence do you feel in talking about sexuality with your ARM patients/their parents?	Patients/parents
1—not at all confident	1 (4.0)/1 (3.6)
2	5 (20.0)/4 (14.3)
3	8 (32.0)/9 (32.1)
4	9 (36.0)/13 (46.4)
5—very confident	2 (8.0)/1 (3.6)
Do you have the feeling that, for your consultation with ARM patients and their parents, you need more support regarding sexual issues?	
Yes	26 (96.3)
No	1 (3.7)
Would you, as a pediatric surgeon, wish to be trained and advised regarding sexuality and sexual issues of ARM patients?	
Yes	21 (77.8)
No	6 (21.4)
Do you think other professionals other than the pediatric surgeon should face sexual issues with ARM patients?	
Yes	26 (92.9)
No	2 (7.1)
Who would be suitable to discuss sexual issues with ARM patients?	
Pediatric surgeons	18 (64.3)
Adult surgeon, expert of ARM	9 (32.1)
Gynecologist/andrologist	27 (96.4)
Sexologist/psychologist	25 (89.3)
Nurse	13 (46.4)
Other^b^	7 (25.0)

*ARM* anorectal malformation

^a^Percentages are calculated from those who responded to the question, so ignoring missing answers

^b^Others mentioned were: pediatric urologist, adult urologist, physiotherapist, pediatrician, multidisciplinary team with the surgeon involved

When we asked about the feeling of confidence and being prepared to talk about sexuality, the opinions of the respondents differed quite a lot, and more than 50% do not feel at all or only moderately confident discussing the topic of sexuality. Women more often (70%) scored a low-to-moderate feeling of confidence compared to men (45%). Five pediatric surgeons (4 males and 1 female) mentioned that their confidence would differ depending on the ARM patient’s gender.

Almost all pediatric surgeons would need support providing consultation on sexual issues to their ARM patients and 78% wish to be trained for that. The majority is of the opinion that other professionals should also face the subject of sexuality of the ARM patients, especially the gynecologists or andrologists, and the psychologists or sexologists.

## Discussion

In 2010, the WHO has defined sexual health as a state of physical, emotional, mental and social well-being, which requires a positive and respectful approach to sexuality as well as sexual relationships and is given the opportunity to enjoy pleasurable and safe sexual experiences that are free of discrimination, coercion, and violence [18]. For attainment and maintaining of sexual health, sexual rights of each individual have to be respected, protected, and fulfilled [18]. Sexual health influences the overall health of an individual and has thus a significant impact on one’s QoL. With the awareness, that evaluation, attainment and maintenance of sexual health are especially lacking in patients with the history of a congenital ARM, the ARM-Net consortium aimed to identify current practice, as well as personal opinions of pediatric surgeons from different European renown pediatric surgical centers towards this specific issue. As demonstrated by a recent review, pediatric surgeons have by now recognized their responsibility to follow their patients with ARM into adulthood [19]. This approach coincides with the fact that more than 90% of the pediatric surgeons who participated in this study follow their ARM patients. However, only half of them screen them for sexual function from neonatal age onwards. This finding reflects that many surgeons are nowadays concerned about urological and bowel function in the postoperative management of ARM, but are less aware of inquiring sexual function as a routine parameter during follow-ups. It has been stated in literature that associated urological and gynecological anomalies may even be missed in the newborn period or at the definitive surgery of ARM, unless the treating surgeon is aware of this association [20, 21].

The majority of female patients with the most complex form of ARM, the cloacal anomaly, was reported to have healthy sexual relationships and may become pregnant [22, 23]. However, recent studies demonstrated that females with ARM experience more frequent sexual dysfunctioning and sexual distress compared to a reference population [7, 8]. Indeed, gynecologic problems during puberty are not uncommon. Pubescent girls with the history of ARM may experience amenorrhea due to an absence or underdevelopment of Müllerian structures or suffer from obstruction to menstrual flow due to a non-patent outflow tract [21]. Further gynecologic problems such as missed vaginal anomalies, hydrosalpinges, adnexal cysts, endometriosis, and chronic pelvic pain associated with endometriosis or pubertal stimulation of chronically adhesed reproductive anatomy may significantly harm female ARM patients during adolescence [21]. Thus, it has been recommended to perform a complete physical examination after puberty that includes an assessment of secondary sexual development and determination of the adequacy of the reproductive anatomy to ascertain sexual intimacy and avoid adverse sequelae [21]. Eventually, parents already would like to learn about the reproductive potential of their child when they are confronted with the diagnosis of ARM, which stresses the need for appropriate counseling of patients and parents to allow optimal sexual development as well as preservation of future fertility.

It is known from literature that males with ARM appear to have a preserved erectile and orgasmic function after being treated by posterior sagittal anorectoplasty, but may suffer from absent ejaculations or azoospermia [8, 24]. In contrast, in male patients treated by abdominoperineal endorectal pull-through (Rehbein’s procedure) or abdominosacroperineal endorectal pull-through for high or intermediate type ARM, the incidence of sexual problems such as erectile and ejaculatory dysfunction was described with a percentage of 40% [25]. These findings also announce the importance to follow patients with ARM into their adulthood, and to evaluate sexual development to provide each individual with ARM, counselling and support when sexual distress occurs.

In this context, this study demonstrates that most pediatric surgeons consider sexuality as an important issue to ARM patients and also do think that it is their responsibility to address questions about sexual issues to their ARM patients. A standardized questionnaire evaluating sexual health in adolescents with ARM is, however, lacking and only a small cohort of pediatric surgeons used specific questionnaires evaluating sexual aspects. Nevertheless, half of the participating pediatric surgeons reported that they ask questions about sexual experiences to their adolescent ARM patients during follow-up visits. On the other hand, many physicians lack the comfort, expertise and willingness to inquire intimate sexual aspects from their patients [15]. This is in alignment with the findings of this study that more than 50% of the pediatric surgeons do not feel at all or only moderately confident discussing the topic of sexuality with their patients and that almost all pediatric surgeons feel that they need more support for consulting their patients and their parents towards sexual issues and actually, wish to be trained in sexuality and sexual issues related to ARM. In fact, it has been stated that physicians feel insecure and reluctant about this vulnerable topic and thus, do not address sexual health proactively as they fear to open a “can of worms” [26, 27]. This may be due to the lack of medical training in human sexuality, which may cause discomfort in talking about sexual issues in doctors [15].

On the other way, it is still infrequent that patients raise a question about sexuality to their surgeon. This finding is most likely linked to the reported hesitancy of patients with chronic illness, which may cause embarrassment and create a barrier to patient–doctor discussion about sex [15]. Furthermore, our findings document that women seem to feel less confident than men in addressing questions about sexuality and that, on the opposite, more men than women believe that their confidence also differs depending on the ARM patient’s gender. Indeed, talking about sexual issues seems to be more comfortable in a same-gender consultation which is preferred by health professionals as well as patients, as demonstrated in a survey from the United Kingdom [26]. Reasons for this preference were stated as concerns that patients of the opposite gender may sexualize the consultation [26]. Apart from that, a tendency that patients self-select health professionals along gender lines was also noted [26]. Thus, health providers should be aware of these problems and find ways to deal with them during the consultation.

In agreement with the wish for a specialized training in sexuality and sexual issues of the majority of pediatric surgeons from this study, it has been recently exclaimed that there is an urgent need for an up-to-date sexual health education not only for present-day physicians, but also for medical students and patients to improve sexual health care [27, 28]. In this context, effective sexual training programs that are designed to build up professional capacity should integrate biological, psychological as well as social aspects of sexuality [28].

In an optimal setting to assess sexual health and manage sexual problems of patients with ARM, pediatric surgeons collaborate with a team of other professions such as gynecologists, andrologists, adult colorectal surgeons, pediatricians, pediatric/adult urologists, psychologists, sexologists, physiotherapists and nurses, which was claimed by the majority of pediatric surgeons. Especially, dedicated psychologists and sexologists with knowledge of ARM may provide substantial support when patients experience sexual dysfunction and dissatisfaction and have problems to start and maintain sexual relationships. Even though, a psychologist, who is in 70% a pediatric psychologist and in 50% dedicated to patients with ARM, is part of the team in most pediatric surgical centers, the possibility to include a sexologist in counselling patients with sexual problems is still uncommon.

One has to keep in mind, that sexual function may be impaired long before a patient with chronic illness reaches puberty, which has an impact on future sexual relationships as well as sexual satisfaction which significantly influences one’s QoL [15]. The ideal age to investigate sexual health during follow-up has been suggested at a mean age of 14.6 years in this study. Breech has recommended to evaluate sexual function in girls after puberty and before the onset of sexual activity, which allows the treating physician to recognize and treat associated urological and gynecological anomalies and their long-term sequelae to enable sexual intimacy [21]. In terms of perceived self-competence during adolescence, having romantic relationships and close friendships in childhood was related to sexual desire and erectile function in male patients with ARM during adulthood, whereas, scholastic competence, physical appearance and global self-worth in childhood negatively predicted sexual distress in female ARM patients during adulthood [8]. These concerns announce the need to address not only physical aspects of sexuality, but also psychosocial and emotional aspects of sexuality during follow-up visits at an adolescent age, which may be best managed in the setting of a transitional outpatient clinic that is involving a multidisciplinary team [8]. In line with the UNESCO guidelines from 2018 [29], educating patients and parents about sexual intimacy should already be encouraged from the age of 5–8 years in an age-appropriate language and way. From a personal view of a patient with ARM from the ARM-Net consortium, this enables the patient to learn about his/her body and accept traumatized parts of the body as their own. This early confrontation with sexual intimacy may eventually help to build up the patient’s body image, but may also create a basis to be able to talk about sexual concerns in a trustful way later on.

In conclusion, this study revealed that pediatric surgeons consider sexuality an important issue to ARM patients and their parents and think that it is their primary responsibility to address questions about sexual issues to their patients. It is of great importance that pediatric surgeons dedicated to ARM provide an adequate continuous support and counselling in concert with a multidisciplinary team, allowing a patient with a congenital ARM to experience “normal” sexual development, achieve sexual health and enjoy sexual relationships later on. To achieve this goal and provide better care, pediatric surgeons who treat patients with ARM shall receive medical training regarding sexual issues and obtain communication skills, to feel more confident in counselling their patients, which is a future objective of the ARM-Net consortium.
